# Systems biology insights into the molecular drivers of childhood stunting and implications for intervention

**DOI:** 10.3389/fnut.2026.1761376

**Published:** 2026-02-25

**Authors:** Genevieve Dable-Tupas, Ariane Blanch A. Maraon, Lorraine Joy L. Bernolo, Nelly Grace F. Toñacao, April Dawn M. Taylaran, Maria Angelica C. Plata, Jason C. Alcano, Richelle D. Björvang, Shamsul Mohd Zain, Vladimer Kobayashi, Melkamu Berhane Arefayine, Alemayehu Teklu Toni, Jacus S. Nacis, Gerard Bryan Gonzales

**Affiliations:** 1 Center for Research and Development, Davao Medical School Foundation Inc., Davao City, Philippines; 2 Department of Public Health and Primary Care, Faculty of Medicine and Health Sciences, Ghent University, Ghent, Belgium; 3 Research and Development Center for Maternal and Child Health (ReDMatCH), Davao Medical School Foundation Inc., Davao City, Philippines; 4 Department of Cellular and Molecular Biology, Uppsala University, Uppsala, Sweden; 5 Department of Pharmacology, Faculty of Medicine, Universiti Malaya, Kuala Lumpur, Malaysia; 6 Rotterdam School of Management, Erasmus University PA, Rotterdam, Netherlands; 7 Department of Mathematics, Physics and Computer Science, College of Science and Mathematics, University of the Philippines Mindanao, Davao City, Philippines; 8 Department of Pediatrics and Child Health, Jimma University, Jimma, Ethiopia; 9 Department of Pediatrics and Child Health, School of Medicine, College of Medicine and Health Sciences, University of Gondar, Gondar, Ethiopia; 10 Human Nutrition and Health, Wageningen University and Research, Wageningen, KD, Netherlands; 11 Department of Science and Technology - Food and Nutrition Research Institute (DOST-FNRI), Taguig, Philippines

**Keywords:** metabolomics, microbiome, mTOR, nutrition, stunting, systems biology

## Abstract

Childhood stunting is a condition resulting from chronic malnutrition affecting millions globally, with lasting consequences for growth, cognition, and productivity. This review explores the molecular mechanisms underlying stunting, focusing on evidence obtained from systems biology to uncover biochemical pathways and potential biomarkers for early detection and targeted interventions. Key findings highlight the role of disrupted pathways such as the mechanistic target of rapamycin (mTOR) signaling, the tryptophan-kynurenine pathway, one-carbon metabolism, and chronic inflammation associated with environmental enteric dysfunction and dysbiosis of the gut microbiome. These insights emphasize the multifactorial nature of stunting, influenced by nutrition, infections, socioeconomic and maternal factors. Integrating systems biology to support public health strategies may provide avenues for precision nutrition-driven interventions that address specific deficiencies and systemic biochemical disturbances.

## Introduction

Childhood stunting is a condition caused by chronic malnutrition which remains a pressing public health issue particularly in low and middle-income countries. It is clinically defined as a height-for-age z-score (HAZ) of less than −2 standard deviation below the global median, as established by the World Health Organization (WHO) (1). Stunting often starts with inadequate weight gain, known as weight faltering. If not properly addressed, this can slow down linear growth over time as the body tries to preserve essential functions by prioritizing basic survival over growth. As a result, height suffers, leading to stunting (2). Apart from directly affecting growth, the consequences of stunting extend far beyond childhood, leading to impaired cognitive and physical development, decreased productivity in adulthood, poor health, and an increased risk of chronic diseases such as diabetes, hypertension, obesity, and metabolic syndrome (1, 3, 4).

Over the past three decades, the prevalence of childhood stunting has declined significantly, from 40% in 1990 to 33% (204 million children) in 2000 and further to 22% (148 million) in 2022 (5, 6). This progress is largely attributed to economic growth, poverty alleviation, targeted nutrition programs, improved water, sanitation, and hygiene (WASH) initiatives, and increased investments in early childhood development (7–9). If the downward trend continues, the number of affected children under five is projected to reach 127 million by 2025. However, this figure remains above the World Health Assembly’s target of 100 million by 2025 (3) and the goal of reducing global stunting prevalence to 13% by 2030 (10). Despite overall progress, disparities persist, with nearly 95% of stunted children in 2022 living in Asia (52%) and Africa (43%) (11).

The global community continues to prioritize stunting reduction, as demonstrated by the World Health Assembly’s nutrition targets and the inclusion of stunting prevention in the Sustainable Development Goals (SDGs) (3, 12, 13). However, progress remains uneven, particularly in low and lower-middle-income countries, where stunting rates remain high at 31% in Africa (14) and between 28 and 45% in South Asia (10).

To fully achieve global nutrition targets, sustained and intensified efforts are essential. While research has primarily focused on nutrition and socioeconomic determinants, molecular-level insights into stunting remain limited. A better understanding of the molecular mechanisms underlying stunting could pave the way for innovative interventions to reduce its prevalence and long-term consequences. This review aims to examine and synthesize the determinants of stunting in children under 5 years old, especially on the molecular perspectives derived from systems biology research.

### Methodology

This review provides a comprehensive synthesis of contemporary literature examining the molecular and metabolic mechanisms underlying childhood stunting, with particular emphasis on systems biology–derived insights. The scope of the review encompasses key biological pathways implicated in growth faltering, including nutrient-sensing signaling (e.g., mTOR), amino acid metabolism, the tryptophan–kynurenine pathway, one-carbon metabolism, inflammation, and their interactions with maternal, environmental, and nutritional factors influencing linear growth and long-term health outcomes.

To ensure consistency and transparency, a structured literature search approach was applied across multiple databases, including PubMed, Scopus, Web of Science, and Google Scholar. For PubMed, Scopus, and Web of Science, search strategies employed combinations of keywords and Boolean operators such as “childhood stunting,” “linear growth faltering,” “systems biology,” “metabolomics,” “mTOR signaling,” “tryptophan-kynurenine pathway,” “one-carbon metabolism,” “amino acid deficiency,” “inflammation,” and “environmental enteric dysfunction.” Titles, abstracts, and keywords were initially screened for relevance, followed by full-text review of articles deemed pertinent to the objectives of this review.

For Google Scholar, the same search terms were used; however, due to limited filtering capabilities, search results were manually screened based on relevance, study quality, and publication date. Priority was given to peer-reviewed human studies, including observational studies, cohort studies, clinical trials, and relevant narrative or systematic reviews, with particular focus on children under 5 years of age and maternal–child dyads.

The literature included in this review primarily spans publications from 2000 to 2025, with earlier seminal studies incorporated where necessary to provide historical or mechanistic context. Collectively, this approach enabled a balanced and integrative evaluation of current evidence linking molecular dysregulation to childhood stunting, while acknowledging the predominantly associative nature of much of the existing data.

#### Contributors and consequences to stunting

Stunting is caused by a complex interaction of biological, socioeconomic, and environmental factors, with maternal health playing a key role. Teenage pregnancy and maternal malnutrition are linked to low birth weight and intrauterine growth restriction (IUGR), which significantly increase the risk of stunting in early childhood (15–18). Male children are biologically more susceptible due to greater energy needs and weaker immune systems (19–21). Children born small for gestational age also face higher risks of infections and poor catch-up growth (22–24).

Furthermore, socioeconomic factors like low parental education limits knowledge of appropriate nutrition, childcare practices, and timely healthcare utilization (25). Inadequate infant feeding practices such as delayed breastfeeding initiation, lack of exclusive breastfeeding, and poor-quality complementary feeding further contribute to growth faltering by increasing infection risk and failing to meet children’s energy and micronutrient requirements (26). Consequently, deficiencies in essential micronutrients and amino acids during critical periods, particularly the first 1,000 days of life, impair growth, immune function, and cognitive development (27, 28). Additionally, environmental exposures, including poor water quality, pollutants, and recurrent infections, also impair nutrient absorption and increase vulnerability to stunting (29–31).

The consequences of stunting are long-lasting and multifaceted, affecting cognitive, educational, economic, and health outcomes. Several studies revealed that stunted children exhibit delays in brain development, learning, and academic achievement, making them 22% more likely not to complete secondary education (32–34). These developmental setbacks reduce employment opportunities and income, with stunted adults earning on average 20% less than their non-stunted peers (35, 36). Families of stunted children also face increased healthcare costs and economic strain (4). On a societal level, stunting diminishes national productivity and economic growth (37, 38). Health-wise, early nutritional deficits alter organ development and metabolic programming, predisposing stunted individuals to chronic diseases such as diabetes, obesity, and hypertension later in life (4, 39, 40).

#### Interventions against stunting

Several studies have explored interventions aimed at reducing childhood stunting with varying results. For instance, interventions such as specialized foods, cash transfers, and behavioral change have shown some level of success in certain regions (41), however these approaches are inconsistent and often fail to address the underlying biological processes that result in stunting. Moreover, traditional methods like micronutrient supplementation alone (42) have not consistently yielded the desired outcomes, and food-based interventions have been limited to improving linear growth without addressing other key outcomes like wasting and poor weight gain (43). Even decades of implementing both nutrition-sensitive and nutrition-specific interventions among rural Gambian children which reduced undernutrition into half, a significant portion (30%) of growth faltering still persists (44, 45).

Furthermore, a systematic review from Ethiopia revealed that two-thirds of the interventions had no measurable effect on stunting (46). These inconsistencies highlight a gap in our understanding of the multifactorial and complex nature of stunting, which is influenced by a combination of factors such as nutritional deficiencies, recurrent infections, socioeconomic conditions, and maternal health. The persistence of stunting despite interventions points to the need for a deeper exploration of these interconnected factors and their contribution to its etiology.

To address these limitations, there is a need to improve understanding in the pathophysiologic mechanisms of stunting. Advanced techniques such as omics technologies offer the potential to identify molecular markers that signal underlying malnutrition, inflammation, or other disruptions in growth pathways.

#### Emergence of systems biology in understanding childhood stunting

Traditional approaches to studying stunting have focused on individual factors such as malnutrition, infections, and socioeconomic conditions. However, these often fail to capture the complex, multifactorial nature of the condition. The emergence of systems biology has provided a transformative approach, integrating multiple biological, environmental, and social determinants to offer a more holistic understanding of the pathophysiology of stunting.

Systems biology is an interdisciplinary field that employs computational and mathematical modeling to analyze biological systems as a whole, rather than in isolated parts (47). In the context of childhood stunting, this approach allows researchers to examine how various factors including genetics, microbiome composition, immune responses, metabolic pathways, and environmental influences, interact to contribute to impaired growth (48). High-throughput omics technologies such as genomics, transcriptomics, proteomics, and metabolomics help to uncover novel biomarkers and mechanistic pathways that underlie stunting (49).

In particular, metabolomics plays a crucial role in systems biology by providing a comprehensive understanding of metabolic perturbations and their connections to genetic, environmental, and pathological factors. Utilizing advanced analytical tools like mass spectrometry and nuclear magnetic resonance, metabolomics enables precise metabolite profiling, bridging genotype and phenotype to decode complex biochemical networks (50). Its integration with other omics fields, such as genomics and proteomics, enhances our ability to study metabolism in diverse contexts, including nutrition and disease. Through these applications, metabolomics strengthens systems biology by offering dynamic insights into metabolic regulation and health outcomes (51).

Furthermore, systems biology has facilitated the development of predictive models and personalized interventions for childhood stunting. By integrating large datasets from diverse populations, machine learning algorithms can identify risk factors and predict stunting trajectories based on early-life exposures (52, 53). This predictive capability enables targeted nutritional and therapeutic interventions, optimizing outcomes for at-risk children. Additionally, systems-based interventions, such as microbiome-targeted therapies (e.g., probiotics, prebiotics, and microbiota-directed complementary foods), are being explored as potential strategies to mitigate the impact of stunting (54).

Systems biology has deepened our understanding of childhood stunting by moving beyond single-factor explanations to a more integrative framework that considers the complex interplay between genetic, microbial, immune, and metabolic factors, albeit many gaps in our knowledge exist especially in translating these findings into effective and scalable interventions.

## Major pathophysiologic mechanisms and biomarkers implicated in stunting

### Perturbation in the mechanistic target of rapamycin complex (mTORC) pathway

The mTOR pathway is a central regulator of cell growth and metabolism, integrating signals from nutrients, energy status, and growth factors. It comprises two complexes: mTORC1 and mTORC2. mTORC1 is sensitive to nutrient levels, especially amino acids, and regulates protein and lipid synthesis, while mTORC2 governs cytoskeletal organization and survival (55, 56).

Amino acids, particularly leucine, are key activators of mTORC1, promoting protein synthesis by phosphorylating downstream targets such as S6K1 and 4E-BP1 (57, 58). Other amino acids like glutamine, arginine, and tryptophan also modulate mTORC1 signaling (59, 60). Disruptions in this signaling cascade have been linked to stunting. Studies in Malawi, Indonesia, and Bangladesh have consistently shown that stunted children have lower circulating levels of essential (e.g., leucine, histidine, methionine) and conditionally essential amino acids (e.g., arginine, glutamine), along with altered lipid metabolites (61–63). This amino acid deficiency likely impairs mTORC1 activity, thereby limiting protein synthesis and growth. In low nutrient conditions, mTORC1 becomes inactive, triggering autophagy to recycle cellular components for survival (64, 65). However, even with sufficient energy and growth factors, mTORC1 cannot function effectively without adequate amino acids (66).

Growth factors such as insulin growth factor 1 (IGF-1) and leptin further modulate mTORC1 through the PI3K/Akt pathway. IGF-1 deficiency has been correlated with stunting in several studies from Bangladesh, Malawi, and Burkina Faso, suggesting long-term endocrine alterations due to early malnutrition (67–69). Another critical hormone, fibroblast growth factor 21 (FGF21), modulates the AMPK-sirtuin-mTOR axis and responds to protein restriction. High baseline FGF21 levels in Bangladeshi children were predictive of better growth outcomes following nutritional supplementation, highlighting its potential as a biomarker for intervention responsiveness (70).

Energy status also plays a critical role in mTORC1 regulation. AMP-activated protein kinase (AMPK), a key energy sensor, inhibits mTORC1 during energy scarcity to conserve resources. Dietary studies in Egypt, Indonesia, and the Philippines reveal that stunted children often consume insufficient energy and protein, which may contribute to mTORC1 suppression (71–73).

**Figure 1:**
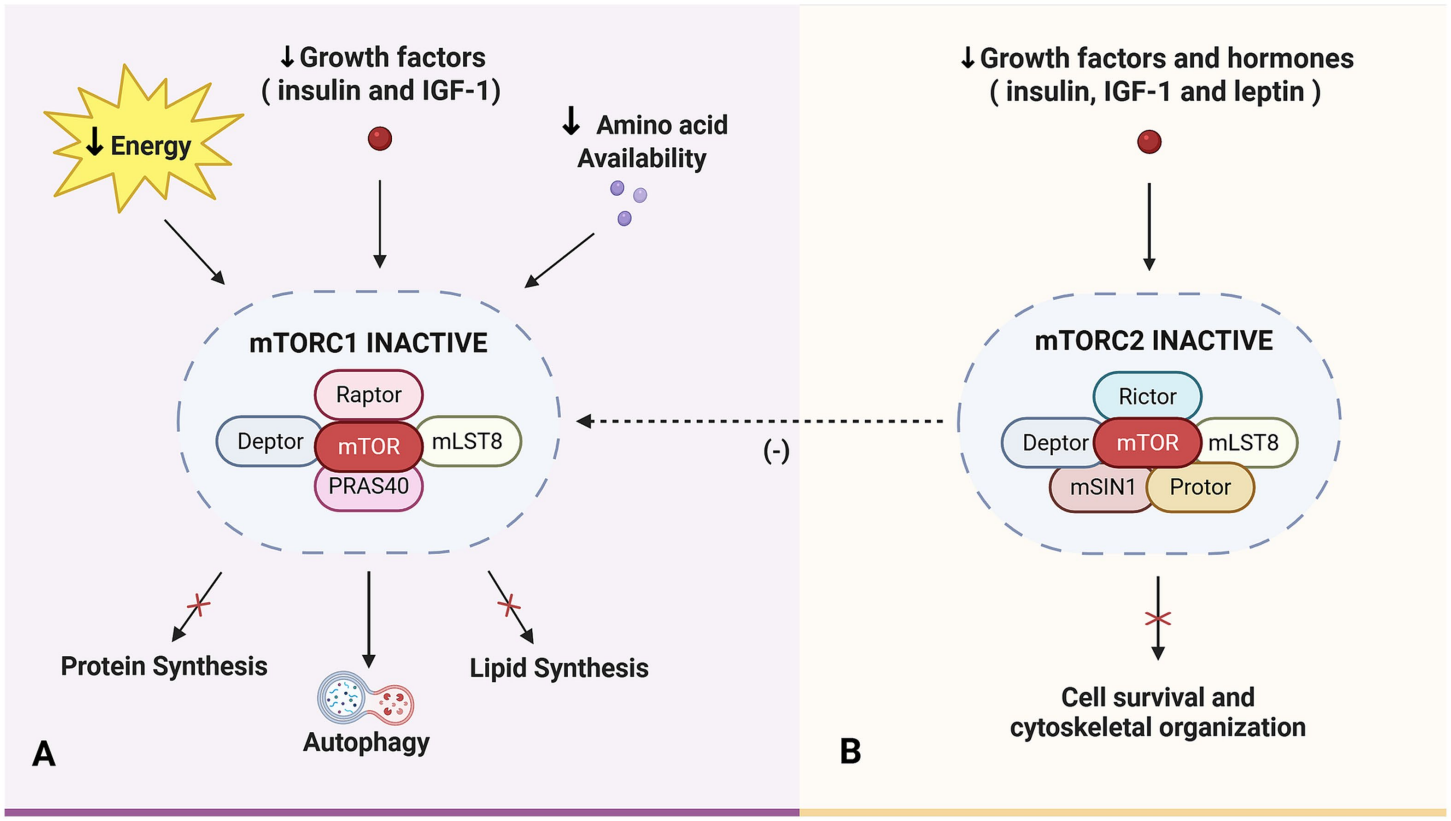
Disruption of mTOR signaling pathways in childhood stunting. **(A)** Reduced availability of amino acids, growth factors, and energy suppresses mTORC1 activity, impairing protein and lipid synthesis and linear growth. **(B)** Reduced hormonal signaling inhibits mTORC2 and secondarily impairs mTORC1 further exacerbating growth deficits. mTOR, Mechanistic Target of Rapamycin; mTORC1, Mechanistic Target of Rapamycin Complex 1; mTORC2, Mechanistic Target of Rapamycin Complex 2; IGF-1, Insulin Growth Factor-1; mLST8, Mammalian Lethal with SEC13 Protein 8; PRAS40, Proline-Rich AKT Substrate of 40 kDa; mammalian stress-activated protein kinase-interacting protein. Created in BioRender. Crd, D. (2026), https://BioRender.com/tbh609p.

mTORC2, while less well-characterized, also contributes to growth regulation. It is activated by insulin, IGF-1, and leptin via PI3K signaling and plays a role in cytoskeletal dynamics and lipid metabolism (74, 75). mTORC2 indirectly enhances mTORC1 activity via Akt-mediated phosphorylation of tuberous sclerosis complex 2 (TSC2) and other downstream targets like PI3KC2-*β* (76, 77). Thus, inhibition of mTORC2 can secondarily impair mTORC1, compounding growth deficits (78). These mechanisms are depicted in Figure 1.

Overall, disruptions in the mTOR pathway whether due to amino acid deficiency, energy deprivation, or hormonal imbalance contribute significantly to the pathophysiology of stunting (Figure 1). Targeting these molecular mechanisms may enhance the efficacy of nutritional and clinical interventions aimed at improving growth outcomes in undernourished children.

Studies supporting the mTOR pathway disruption are summarized in Table 1.

**Table 1:** Summary of the relevant studies supporting the dysregulation of the mTOR pathway leading to growth faltering changes in childhood stunting.

Population	Type of study (Method)	Biomarkers/key findings	References
0–35 months admitted malnourished children in Burkina Faso (*n* = 59)	Cohort study (IGF-1 Assay from dried blood spots)	IGF-1 levels from capillary blood samples significantly increased after nutritional rehabilitation and correlated with weight-for-height Z-score changes.	Kouanda et al., 2009 (69)
Children in rural southern Malawi aged 12–59 months (*n* = 313)	Cross-sectional Targeted (Metabolomics and Lipidomics)	Stunted children in rural Malawi had low serum levels of essential amino acid and sphingolipids compared with non-stunted children. Children with a high risk of stunting may not be receiving an adequate dietary intake of essential amino acids and choline. Stunted children had lower serum sphingomyelins	Semba et al., 2016 (61)
Children from different countries (Malawi, Bangladesh, and Sweden; *n* = 50; 25 stunted & 25 normal children)	Genome-scale metabolic profiling	Stunted children had reduced plasma levels of essential amino acids as well as lower ratio of tryptophan to other neutral amino acids compared to the healthy group	Kumar et al., 2018 (63)
6–13 months underweight children in Dhaka, Bangladesh (*n* = 120)	Prospective cohort study (ELISA)	Plasma FGF21 levels showed a negative association with changes in WAZ and LAZ. However, underweight children with initially high FGF21 levels, had higher WAZ and LAZ, suggesting better response to nutritional supplementation.	Arndt et al., 2019 (70)
Children treated for severe acute malnutrition (SAM; *n* = 352)	Cohort study (Tandem mass spectrometry, NMR and ELISA)	Stunted children with SAM showed low plasma IGF-1 levels	Bourdon et al., 2019 (68)
Children in the slums of Bangladesh aged between 12 and 18 months (*n* = 100; 50 stunted & 50 normal children)	Quasi-experimental study (ELISA)	Serum leptin, leptin–adiponectin ratio, IGF-1, and IFN-γ were independently associated with stunting in Bangladeshi children under the age of two.	Hossain et al., 2019 (67)
Indonesian children aged 25–30 months (*n* = 121; 36 stunted & 85 normal)	Case–control study	A significantly higher percentage of stunted children (30.6%) had protein intake below the recommended level compared to normal children (8.2%)	Fikawati et al., 2021 (72)
Indonesian children aged to 24–59 months (*n* = 80; 23 stunted & 57 normal children)	Descriptive, Case–control study	Stunted children may not receive sufficient dietary intake of EAAs in their diet.	Rizky & Sutjiati, 2021 (62)
Preschool children (2-5yo) in rural Egypt (*n* = 497)	Community-based cross-sectional study	Stunted children consumed poultry, eggs, and fruits significantly less frequently than their non-stunted counterparts.	Mahfouz et al., 2022 (71)
5–10-yr-old school-age Filipino children (*n* = 26,332)	Retrospective study	Stunted school-age children had significantly lower intake of energy, protein, and key micronutrients, including vitamin A, vitamin C, thiamin, niacin, riboflavin, iron, and calcium, compared to their non-stunted peers	Arias et al., 2024 (73)

mTOR, Mechanistic target of rapamycin; EAA, Essential amino acids; IGF-1, Insulin growth factor-1; IFN-γ, Interferon gamma; ELISA, Enzyme linked immunoassay; WAZ, Weight for age z-score; LAZ, Length for age z-score; FGF21, Fibroblast growth factor 21.

### Tryptophan-kynurenine pathway dysregulation

The relationship between the tryptophan-kynurenine pathway (TKP) and childhood stunting is complex and influenced by chronic inflammation, nutritional deficiencies, and metabolic alterations. Tryptophan is an essential amino acid which plays a significant role in protein synthesis and serves as a precursor of two main pathways: the serotonin pathway and the kynurenine pathway. Under normal physiological conditions, most dietary tryptophan (over 90%) is metabolized via the kynurenine pathway, while only about 1% is used for serotonin synthesis, which serves a critical role in cellular function. The rest of the unmetabolized tryptophan is used for protein synthesis in tissues like muscles. This metabolic balance is generally maintained unless disrupted by inflammation or stress, which can shift tryptophan metabolism away from serotonin and protein production, leading to physiological and neurological consequences (79–81).

In the serotonin pathway, tryptophan is first hydroxylated by tryptophan hydroxylase-1 (TPH-1), the rate-limiting enzyme in serotonin biosynthesis, producing 5-hydroxytryptophan (5-HTP), which is then converted into serotonin (79, 82). Additionally, the availability of amino acids, including tryptophan, activates the mTORC1 pathway, a growth regulator that promotes protein synthesis which is essential for child growth (83). However, chronic inflammation can significantly alter this process by upregulating the kynurenine pathway, diverting tryptophan metabolism toward kynurenine synthesis. This shift is driven by increased activity of indoleamine 2,3-dioxygenase 1 (IDO1), the key enzyme in the kynurenine pathway, whose expression is elevated in inflammatory conditions (79, 84, 85). In addition, tryptophan 2,3-dioxygenase (TDO), a liver-specific enzyme, also catalyzes the first step of tryptophan degradation under homeostatic conditions and in response to glucocorticoids and tryptophan levels, further regulating systemic tryptophan availability and influencing the balance between serotonin and kynurenine pathway metabolism (86).

**Figure 2:**
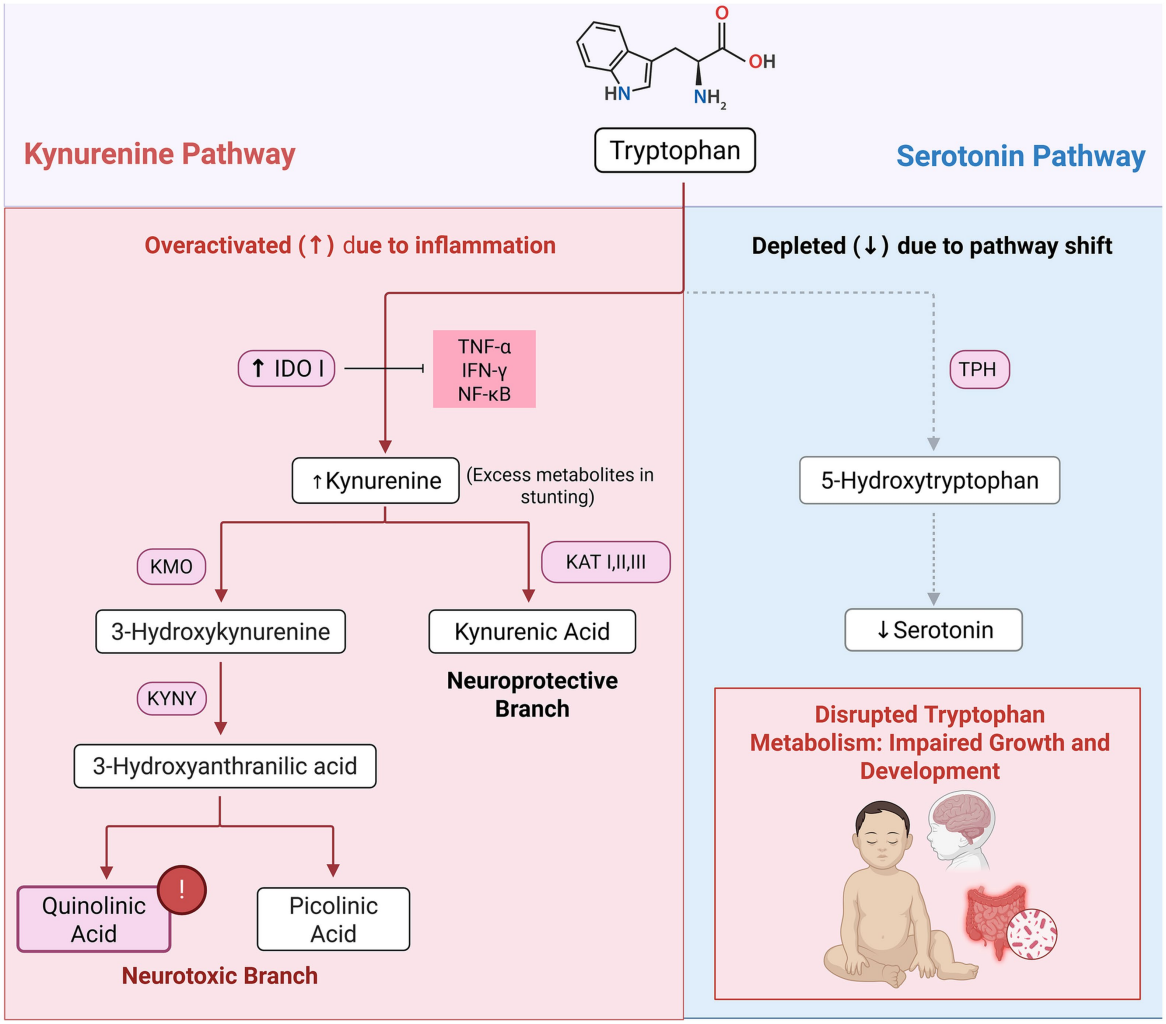
Tryptophan-kynurenine pathway dysregulation resulting in impaired growth and neurodevelopment. Systemic and intestinal inflammation increase pro-inflammatory cytokine levels (TNF-*α*, IFN-*γ*, and NF-κB), which activate IDO1, diverting tryptophan toward the kynurenine pathway and its metabolites. This metabolic shift (dotted arrow) reduces tryptophan availability, leading to decreased serotonin production and contributing to impaired growth and cognitive development. TNF-α, Tumor Necrosis Factor-α; IFN-γ, Interferon-γ; NF-κB, Nuclear Factor kappa-B; IDO, indoleamine 2,3-dioxygenase; KMO, Kynurenine 3-monooxygenase; KAT I, Kynurenine aminotransferase I; KAT II, Kynurenine aminotransferase II; KAT III, Kynurenine aminotransferase III; KYNU, Kynureninase; TPH, Tryptophan hydroxylase. Created in BioRender. Crd, D. (2026), https://BioRender.com/i15r554.

In the context of childhood stunting, chronic inflammation commonly associated with environmental enteric dysfunction, elevates pro-inflammatory cytokines such as Tumor Necrosis Factor-*α* (TNF-α), Interferon-*γ* (IFN-γ), and Nuclear Factor kappa-B (NF-κB). These inflammatory signals activate IDO1, increasing the conversion of tryptophan to kynurenine and its downstream metabolites, thereby reducing tryptophan availability for serotonin synthesis and protein production (86–88). This metabolic shift contributes to abnormally low serotonin and protein levels, impairing both linear growth and cognitive development (Figure 2) (85, 87). Gazi et al. (83) reported that elevated kynurenine-to-tryptophan (K/T) ratios indicating increased tryptophan catabolism, are negatively associated with linear growth. Chronic inflammation not only accelerates catabolism in kynurenine pathway but divert tryptophan away from serotonin and protein production leading to a metabolic imbalance which contributes to muscle wasting and weight loss and far-reaching consequences on cognitive development and growth (87, 89, 90).

Metabolomics studies demonstrated a relationship between altered tryptophan metabolism and childhood stunting. Guerrant et al. (91) found that lower plasma tryptophan levels correlate with biomarkers of systemic and intestinal inflammation in Brazilian children, reinforcing the association between tryptophan depletion and growth impairment. Similarly, Kosek et al. (90) reported that low plasma tryptophan levels are linked to growth deficits in impoverished children. Their analysis of child cohorts in Peru and Tanzania revealed a direct correlation between plasma tryptophan concentrations and linear growth up to 8 months after biomarker assessment. Conversely, increased kynurenine production due to increased IDO1 activity is associated with intestinal injury and inflammation, ultimately leading to poorer growth outcomes.

Dietary intake also plays a crucial role in tryptophan metabolism. In Tanzania, for instance, a maize-based diet has been linked to low tryptophan intake. Even as low as 25% below the required tryptophan intake can reduce the synthesis of proteins leading to symptoms such as anorexia and impaired growth (83, 90). This further stresses the importance of tryptophan as a critical biomarker for growth and nutritional status.

In stunted children, the combination of low tryptophan availability and heightened kynurenine production exacerbates the effects of malnutrition and inflammation, creating a detrimental cycle that impairs growth and development (Figure 2) (61).

Additionally, kynurenine and its derivatives exhibit complex immunomodulatory effects, influencing immune responses by promoting T-cell apoptosis and inhibiting T-cell proliferation. These immunosuppressive effects can exacerbate the impact of infections prevalent in stunted populations, creating a vicious cycle where chronic inflammation increases tryptophan catabolism, further compromising immune function and nutrient absorption, ultimately resulting in stunted growth (83, 92, 93).

It should be noted, however, that in the Sanitation Hygiene Infant Nutrition Efficacy (SHINE) trial conducted in Zimbabwe, the K/T ratio was significantly associated with stunting only at 12 months of age, introducing some inconsistency across studies (94). Nevertheless, the majority of evidence supports a strong association between dysregulation of the TKP and childhood stunting, with elevated kynurenine levels proposed as potential biomarkers of growth impairment. A summary of the relevant studies and their key findings is presented in Table 2.

**Table 2:** Summary of the relevant studies supporting the impairment of the tryptophan-kynurenine pathway among children.

Population	Type of study (Method)	Biomarkers/key findings	References
Malnourished and Normal Children from Brazil aged 6–26 months (*n* = 375)	Case–control (Targeted Metabolomics)	Lower plasma tryptophan levels correlate with biomarkers of intestinal and systemic inflammation. Tryptophan depletion is linked to compromised growth.	Guerrant et al., 2016 (91)
Newborns less than 17 days of age in rural Peru and Tanzania who were >1,500 g at birth (*n* = 494)	Cohort (Targeted Metabolomics)	Plasma tryptophan concentrations are inversely associated with the development of statural growth deficits in children. Elevated plasma K/T ratios are negatively associated with linear growth.	Kosek et al., 2016 (90)
Children aged 12–59 months from rural Malawi (*n* = 313)	Cross-sectional (Targeted metabolomics)	Children with stunting had lower serum concentrations of tryptophan.	Semba et al., 2016 (61)
Bangladeshi children aged between 12 and 18 months who are stunted or at risk of stunting (*n* = 480)	Community based interventional study (Targeted Metabolomics)	High kynurenine levels are linked to poor cognitive and linear growth High plasma K/T ratio was found to be significantly and negatively associated with linear growth.	Gazi et al., 2020 (83)
SHINE Trial in Zimbabwe Mother-infant dyad (*n* = 1,169 infants)	Cluster randomized trial (UHPLC–MS/MS)	An elevated K/T ratio at 12 months was associated with a decrease in mean LAZ velocity.	Mutasa et al., 2021 (94)

K/T, Kynurenine-to-tryptophan; LAZ, Length for age z-score; SHINE, Sanitation Hygiene Infant Nutrition Efficacy; UHPLC–MS/MS, Ultra-high-performance liquid chromatography–tandem mass spectrometry.

### Dysfunction of one-carbon metabolism or methylation pathways

**Figure 3:**
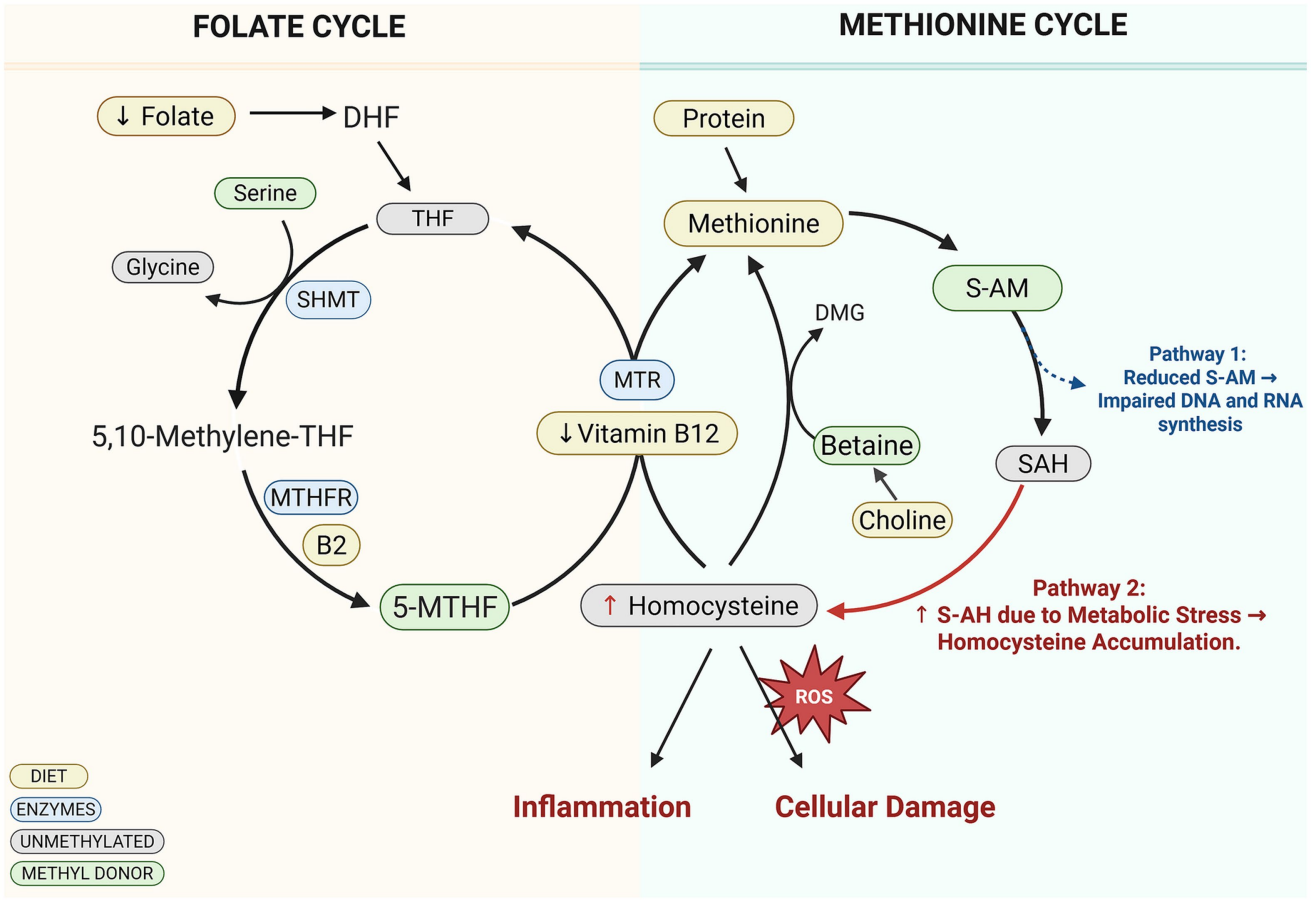
Dysfunction of one-carbon metabolism leads to inflammation, cellular damage, and impaired DNA and RNA synthesis contributing to growth faltering changes. Decrease in folate and/or B vitamins causes dysfunction of one carbon metabolism adversely affecting both folate and methionine cycles leading to increased homocysteine levels and related metabolites resulting in cellular damage and/or inhibition of DNA and RNA synthesis contributing to growth impairment. DNA, Deoxyribonucleic acid; RNA, Ribonucleic acid; DHF, Dihydrofolate; THF, Tetrahydrofolate; SHMT, Serine hydroxymethyltransferase; MTHFR, Methylenetetrahydrofolate reductase; B2, Riboflavin (vitamin B_2_); 5-MTHF, 5-Methyltetrahydrofolate; MTR, Methionine synthase; DMG, Dimethylglycine; S-AM, S-Adenosylmethionine; SAH, S-Adenosylhomocysteine. Created in BioRender. Crd, D. (2026), https://BioRender.com/s65j5t4.

One-carbon metabolism (OCM) plays a vital role in early development by providing one-carbon units necessary for the synthesis of deoxyribonucleic acid (DNA), proteins, and lipids, as well as for epigenetic modifications that regulate gene expression (95). This interconnected network, which includes the folate and methionine cycles, acts as an integrator of nutrient status and relies on essential nutrients such as B vitamins, amino acids, choline, betaine, and methionine for proper function (96, 97). These nutrients drive critical biochemical reactions that support DNA replication, repair, and methylation (98, 99). Functional biomarkers like S-adenosylmethionine (S-AM) and homocysteine further reflect OCM’s role in maintaining cellular health and epigenetic regulation (100). Given its significance during pregnancy and childhood, impairments in OCM have been closely linked to stunting and malnutrition (Figure 3) (101).

The role of OCM in childhood malnutrition and growth impairment is increasingly evident, as deficiencies in key nutrients disrupt methylation processes critical for gene regulation and cellular function (102). Studies have shown that children with edematous severe acute malnutrition, such as kwashiorkor, exhibit widespread DNA hypomethylation compared to those with non-edematous Severe Acute Malnutrition (SAM), likely due to low methionine levels and reduced methylation capacity—changes that are reversible with nutritional rehabilitation (103). In Malawian children with kwashiorkor, significantly lower serum levels of methionine, homocysteine, and related metabolites further support the link between OCM dysfunction and redox (101).

Epigenetic alterations such as elevated histone H3 lysine 9 trimethylation have also been observed in stunted children and are associated with suppressed immune gene expression and impaired linear growth from birth to 1 year (104). Notably, these molecular changes were detected prior to the clinical manifestation of stunting, highlighting their potential as early biomarkers of growth faltering.

Maternal deficiencies in one-carbon nutrients such as folate, vitamin B6, and B12 are linked to elevated homocysteine levels and adverse pregnancy outcomes, including fetal growth restriction, low birth weight, and preterm birth (105, 106). Adequate intake of these nutrients is crucial for regulating homocysteine metabolism and supporting healthy fetal development. A study among Chinese pregnant women revealed significant imbalances in OCM biomarkers during mid-to-late pregnancy, including low levels of folate and B vitamins and elevated total homocysteine. Elevated homocysteine was inversely associated with red blood cell folate and vitamin B6 levels, while plasma S-AM showed a positive relationship with serum betaine and a negative one with vitamin B6 (97). These findings emphasize the critical need to ensure sufficient one-carbon nutrient levels during pregnancy for optimal maternal and fetal health.

Stunted children have shown reduced levels of choline-derived metabolites such as betaine and dimethylglycine (DMG), which are vital for growth-related processes like cell proliferation and gene regulation (107–110). Studies in Malawian children found significantly lower serum choline and phosphatidylcholine levels among those who were stunted, alongside higher betaine-to-choline and trimethylamine N-oxide (TMAO)-to-choline ratios—patterns associated with impaired growth (61, 111). In Brazilian children, urinary excretion of choline and DMG was positively linked to better growth outcomes (112). Furthermore, a longitudinal lipidomics study of a birth cohort in Gambia identified serum polyunsaturated fatty acids (PUFAs) and phosphatidylcholines as reliable predictors of future growth, highlighting their importance in early-life dietary interventions (113).

Interventions targeting PUFAs and choline, particularly through egg consumption, have shown mixed results in reducing childhood stunting. The 2014 Lulun Project demonstrated that consuming one egg daily for 6 months during early complementary feeding reduced stunting by 47% and increased linear growth by 0.63 length-for-age Z-score (LAZ). This intervention significantly elevated plasma levels of choline, betaine, methionine, TMAO, dimethylamine (DMA), and docosahexaenoic acid (DHA), which are key components in metabolic and growth-related processes (114).

However, a follow-up study, Lulun Project II, tracking over 90% of the original cohort, found that the growth benefits were not sustained beyond 2 to 3 years of age. HAZ declined more in the egg group than in the control group, indicating greater growth faltering over time. These findings suggest that while egg consumption provides crucial early benefits, longer intervention periods and a more comprehensive approach to stunting prevention are needed (115).

Other studies report varying results. In Ethiopia, Omer et al. (116) found that a child-owned poultry intervention significantly reduced stunting rates and improved nutritional status in children aged 6–18 months. However, Stewart et al. (117) reported that a six-month egg intervention did not improve linear growth among Malawian children. Similarly, Ricci et al. (118) found no significant improvement in linear growth or other health parameters in South African children following a six-month egg intervention.

Despite these inconsistencies, a meta-analysis of seven egg intervention trials concluded that overall, egg consumption was associated with improved height outcomes in children (119). These findings highlight the potential role of eggs in child growth but also suggest the need to consider additional dietary and environmental factors for long-term effectiveness (Table 3).

**Table 3:** Summary of studies supporting one-carbon metabolism dysfunction as contributory to growth faltering changes in childhood stunting.

Population	Type of study (Method)	Biomarkers/key findings	References
Children from Ceará, Brazil, 6–24 months of age (*n* = 326)	Case–Control [1H nuclear magnetic resonance (NMR) spectroscopy]	Stunted children excreted lower levels of betaine and DMG in their urine.	Mayneris-Perxachs et al., 2016 (108)
Malawian Children 12–59 months of age (*n* = 325)	Cross-Sectional (Metabolomics)	Lower serum choline; higher betaine-to-choline and TMAO-to-choline ratios in stunted children	Semba et al., 2016 (111)
Lulun Project I: Infants aged 6–9 mos in Ecuador (*n* = 148)	Randomized controlled trial (Chemilumines-cent competitive immunoassay; Metabolomics)	The egg intervention significantly increased plasma choline, betaine, methionine, TMAO, DMA, and DHA. One egg per day for 6 months during early complementary feeding reduced stunting by 47% and increased linear growth by 0.63 length-for-age Z score.	Iannotti et al., 2017 (114)
Children from Malawi and Jamaica between 6 and 59 months of age (*n* = 309 children)	Cohort (Genome-wide DNAmethylation analysis)	Significant DNA hypomethylation at 877 CpG sites (99% hypomethylated) in ESAM compared to NESAM Low methionine levels and reduced methylation activity contributes to hypomethylation.	Schulze et al., 2019 (103)
Biological samples from infants in the Peru (*n* = 281), Bangladesh (*n* = 249), and Tanzania (*n* = 249) sites of the MAL-ED birth cohort	Cohort (Nuclear Magnetic Resonance Spectroscopy)	Stunted children in Malawi had lower choline levels compared to their non-stunted peers. Brazilian children: choline and DMG urinary excretion showed a positive correlation with growth Betaine highest demand is in the first 6 months of life	Giallourou et al., 2020 (112)
Lulun Project II: Follow-up study of the Lulun Project I approximately 2-year timeframe (*n* = 135)	Cohort Study (Metabolomic analysis of blood biomarkers was not carried out)	Over 90% of children successfully completed the Lulun Project’s original trial. The egg intervention’s effect was no longer present in children aged 2–3 years. Significant declines in HAZ were observed in the egg group compared to the control group.	Iannotti et al., 2020 (115)
Gambian Children 3 months of age up to 2 years (*n* = 409)	Cohort Longitudinal (five time points; Lipidomics)	Lipid groups with PUFAs and phosphatidylcholines predict future growth outcomes. Lipids had stronger association to height than weight, suggesting higher nutritional demand for height. PUFAs and choline are crucial in early dietary interventions to prevent growth faltering in low-income settings.	Gonzales et al., 2021 (113)
Malawian Children between the ages of 12 and 60 months (*n* = 422 children)	Cross-sectional (Metabolomics)	Significantly lower serum levels of methionine, homocysteine, cystathionine, cysteine, and asymmetric dimethylarginine in children with kwashiorkor and marasmic-kwashiorkor	May et al., 2022 (101)
Southern Ethiopian Children 6–18 months old (*n* = 243)	Cluster-randomized community trial (Intestinal helminthiasis examination)	Nutrition-sensitive poultry intervention improved children’s nutritional status and gross motor milestone development. Significant increase in weight-for-age and weight-for-height Z-scores.	Omer et al., 2022 (116)
18-week old children, and mothers in Dhaka, Bangladesh (*n* = 29; 15 infants & 14mothers)	Cohort (Epigenetic Profiling)	Globally elevated H3K9me3 levels were associated with poor linear growth between birth and 1 year of age. H3K9me3 changes were detectable before the overt appearance of the stunted phenotype, suggesting potential as early biomarkers.	Kupkova et al., 2023 (104)
Children 6 months to 18 years old (*n* = 3,575)	Metaanalysis of 7 egg intervention studies	Participants in the egg intervention groups showed significantly greater increase in height/length and weight compared to those in the control groups.	Larson et al., 2024 (119)
Pregnant women at 24–32 gestational weeks having single pregnancy (*n* = 397)	Cohort (Metabolomics and Immunoassay)	Imbalance in blood OCM during mid-to-late pregnancy: lower folate, B6, B12, and elevated total homocysteine (tHcy) Adequate folate and B6 are significant predictors of lower tHcy. Higher serum tHcy is linked to lower RBC folate and vitamin B6 Higher plasma S-AM is positively associated with serum betaine and negatively with vitamin B6.	Zhang et al., 2024 (97)

ESAM, Edematous severe acute malnutrition; NESAM, Non edematous severe acute malnutrition; DNA, Deoxynucleic acid; H3K9me3, H3 lysine 9 trimethylation; tHcy, total homocysteine; PUFAs, Polyunsaturated fatty acids; DMG, Dimethylglycine; MAL-ED, Etiology, Risk Factors, and Interactions of Enteric Infections and Malnutrition and the Consequences for Child Health and Development; TMAO,Trimethylamine N-oxide; DMA, Dimethylamine; DHA, Docosahexaenoic acid; HAZ, Height for age z-score.

### Chronic inflammatory pathway, environmental enteric dysfunction and the role of the microbiome

Inflammation is a normal immune response to harmful stimuli. However, chronic inflammation disrupts the balance of immune signaling, leading to oxidative stress, tissue damage, and metabolic disturbances that can negatively affect child growth (120). Elevated pro-inflammatory cytokines like TNF-*α* and interleukin 6 (IL-6) have been linked to impaired nutrient absorption, hormonal dysregulation, and reduced energy availability. However, findings on TNF-α levels in stunted children vary: Zambruni et al. (121) observed elevated TNF-*α* among stunted Peruvian infants, whereas Nuryandari et al. (122) and Hossain et al. (67) reported lower levels among older stunted children with chronic infections. These discrepancies may reflect differences in age, health status or immune suppression due to severe malnutrition and wasting (123). Supporting this, other studies show that TNF-α levels correlate with body mass index (BMI), suggesting that lower TNF-α may be a marker of severe malnutrition and immune dysfunction (124–126).

One potential driver of chronic inflammation and growth impairment is EED, a subclinical condition prevalent in low and middle-income countries. EED arises from repeated exposure to enteric pathogens due to poor sanitation and hygiene and is characterized by chronic intestinal inflammation, villous blunting, crypt hyperplasia, increased intestinal permeability, and impaired nutrient absorption (29).

EED contributes to growth faltering through multiple, interrelated mechanisms. Recurrent pathogen exposure (e.g., *Escherichia coli* and Shigella) induces sustained production of inflammatory cytokines such as TNFα and IL6, which interfere with growth hormone signaling, suppress IGF-1, and divert energy toward immune responses. TNF α–mediated activation of the NF κB pathway further amplifies inflammation and inhibits anabolic processes required for linear growth (127). Intestinal damage in EED, such as villous atrophy and crypt hyperplasia, impairs nutrient absorption and increases gut permeability, allowing bacterial products to enter the bloodstream and sustain systemic inflammation. Humphrey (128) highlighted how this “leaky gut” phenomenon triggers an immune response that diverts nutrients and energy away from growth processes, exacerbating stunting. Prendergast et al. (129) further demonstrated that chronic inflammation suppresses the IGF-1 axis, leading to hormonal disruptions that impair linear growth. Harper et al. (130) observed that children with EED exhibited poor HAZ due to persistent intestinal damage. A recent systematic review demonstrated that all EED domains including intestinal damage and repair, absorption and permeability, microbial translocation, intestinal inflammation, and systemic inflammation are consistently associated with impaired linear growth in children (131).

The gut microbiome plays a central role in mediating these effects. Metagenomic studies consistently show that stunted children exhibit an immature and dysbiotic gut microbiome, which compromises nutrient utilization, immune regulation, and metabolic signaling (132). This dysbiotic state alters endocrine pathways critical for growth, including the IGF-1 axis (133).

Microbiome-derived metabolites further link gut dysfunction to stunting. Short-chain fatty acids (SCFAs), particularly butyrate, support epithelial integrity, modulate immune responses, and provide energy to colonocytes (134). Stunted children show reduced abundance of butyrate-producing taxa such as Faecalibacterium, Megasphaera, and Blautia, alongside increased Ruminococcus, a pattern associated with intestinal inflammation and barrier dysfunction (135). Data from the Etiology, Risk Factors and Interactions of Enteric Infections and Malnutrition and the Consequences for Child Health and Development (MAL-ED) cohort indicate that subclinical, non-diarrheal infections with Shigella, enteroaggregative *Escherichia coli*, Campylobacter, and Giardia are associated with larger declines in length-for-age Z-scores than infections caused by other microbes (136). Giardia infection has additionally been linked to amino acid deficiencies and elevated phenolic acids, reflecting altered microbial amino acid metabolism (137).

Disruption of tryptophan metabolism represents another microbiome-mediated pathway. Gut dysbiosis can shift tryptophan metabolism toward the kynurenine pathway, promoting inflammation and impairing gut barrier function (138). Reduced levels of indole-3-propionic acid, a microbiota-derived tryptophan metabolite, have been associated with epithelial damage and intestinal inflammation in EED (139).

Bile acid metabolism is also markedly altered in stunted children. Dysbiosis disrupts bile acid composition and enterohepatic circulation, impairing lipid absorption and immune regulation. Reduced duodenal concentrations of secondary bile acids such as deoxycholic and lithocholic acid (140–142), alongside elevated plasma glycine-conjugated bile acids, suggest bile acid malabsorption and contribute to diarrhea, systemic inflammation, and growth impairment (143). In addition, microbial fermentation of undigested substrates generates inflammatory products such as lipopolysaccharides, which are elevated in stunted children and further activate immune pathways (91, 144). A summary of these studies is provided in Table 4.

**Table 4:** Summary of studies supporting the role of chronic inflammation, environmental enteric dysfunction and the role of the microbiome in growth impairment among children.

Population	Type of study (Method)	Biomarkers/key findings	References
Malnourished and Normal Children from Brazil aged 6–26 months with 2–6 months follow up (*n* = 375)	Case Control (ELISA)	Children <12 months: ↑ plasma IgA, LPS, FliC, and Intestinal-FABP levels; Children >12 months: increased plasma zonulin suggests prior intestinal barrier disruption. Stunted children showed reduced SAA, indicating weakened host defense, while higher citrulline and tryptophan levels reflected a systemic response to intestinal disruption and inflammation.	Guerrant et al., 2016 (91)
MAL-ED longitudinal birth cohort (*n* = 1469)	Multi-site Cohort (Quantitative PCR)	Subclinical non-diarrheal infection with Shigella, enteroaggregative *Escherichia coli*, Campylobacter and Giardia showed larger decrease in LAZ than other microbes	Rogawski et al., 2018 (136)
Infants aged 5–12 months and followed up for 6 months from Peru (*n* = 78)	Pilot Prospective Cohort (ELISA and Metagenomics)	Elevated serum I-FABP, TNF-α, and CD14 levels Ruminococcaceae (Ruminococcus 1 and 2) and Coriobacteriaceae (Collinsella) increased over time in stool of children who became stunted Decrease in the relative abundance of one genus of Enterobacteriaceae (Providencia)	Zambruni et al., 2019 (121)
Children in the slums of Bangladesh aged between 12 and 18 months (*n* = 100; 50 stunted & 50 normal children)	Quasi-experi-mental (ELISA)	Decreased blood leptin production in stunted children prior to intervention (food supplementation and psychosocial stimulation) Levels of blood CRP and most of the pro-inflammatory cytokines (IL-6, IL-12, and TNF-α,) were lower among stunted children	Hossain et al., 2019 (67)
MAL-ED longitudinal birth cohort (*n* = 1469)	Multi-site Cohort (MAL-ED cohort and a novel gnotobiotic murine model)	Giardia infection is associated with stunting among children with amino acids deficiencies with over production of phenolic acids	Giallourou et al., 2023 (137)
Stunted Children 0–5yo with Chronic Infection from Indonesia (*n* = 48)	Cross-sectional (ELISA)	Lower blood mean level of IGF-1 and TNF-α level in stunted children with chronic infection	Nuryandari et al., 2024 (122)
Pakistan EED Cohort (*n* = 52); Zambia EED Cohort (*n* = 30); USA Normal Controls (*n* = 25)	In-silico metabolic network modeling (Multi-omics)	Increased phosphatidylcholine, lysophosphatidylcholine (LPC) and ether-linked LPCs, and decreased ester-linked LPCs were observed in the duodenal lipidome of Pakistan EED subjects Plasma levels of glycine-conjugated bile acids were significantly increased.	Zulqarnain et al., 2024 (141)
Stunted versus non-stunted children under 5 years in LMICs (Metaanalysis was not done)	Systematic Review of 14 studies (Genomic Sequencing)	No difference in alpha diversity Higher beta diversity in stunted children Abundance of pro-inflammatory Escherichia/Shigella and Campylobacter; ↓butyrate producers and ↑ Ruminococcus	Chibuye et al., 2024 (135)
Children 0–5 years in LMIC	Systematic Review of 80 studies from 31 countries (Observational and Interventional)	Biomarkers of EED related to intestinal inflammation, permeability, and microbial translocation are associated with impaired linear growth Elevated fecal inflammatory markers (myeloperoxidase and calprotectin), markers of gut permeability (including lactulose:mannitol ratio), and systemic inflammation markers are frequently linked to stunting.	Lowe et al., 2025 (131)

SAA, Serum amyloid A; ELISA, Enzyme linked immunoassay; IGF-1, Insulin growth factor-1; I-FABP, Intestinal fatty acid-binding protein; TNF-α, Tumor necrosis factor-alpha; MAL-ED, Etiology, Risk Factors and Interactions of Enteric Infections and Malnutrition and the Consequences for Child Health and Development.

Environmental and nutritional factors strongly modulate EED risk. Poor sanitation, inadequate hygiene, and close contact with livestock increase exposure to enteric pathogens, while nutrient-poor diets limit intestinal repair and immune competence (145). Observational studies consistently show that children living in unhygienic environments or households practicing open defecation exhibit higher EED biomarker levels and poorer growth outcomes (146) found that children exposed to unsanitary environments exhibited elevated markers of gut inflammation and stunted growth. Although improvements in environmental hygiene are associated with reduced intestinal inflammation (147), evidence from randomized trials indicates that conventional household-level water, sanitation, and hygiene (WASH) interventions alone have limited effects on EED biomarkers and stunting (148, 149). Large trials in Bangladesh, Kenya, and Zimbabwe demonstrated that while improved infant and young child feeding (IYCF) enhanced linear growth, household-level WASH interventions did not consistently reduce stunting or enteropathogen exposure (150). Recent systematic reviews confirm that poor WASH conditions are strongly associated with elevated EED biomarkers, yet WASH interventions show inconsistent effects, highlighting the need for transformative, community-level and nutrition-integrated approaches (131, 151, 152).

Dietary inadequacies further exacerbate EED and growth failure. Deficiencies in key micronutrients, particularly zinc (153–155) and iron (156, 157), impair epithelial repair, weaken immune defenses, and intensify chronic inflammation, compounding the effects of environmental exposures.

Collectively, current evidence indicates that stunting associated with chronic inflammation and EED arises from complex interactions among enteric infections, gut dysbiosis, metabolic dysfunction, and nutritional deficiencies. Effective prevention and mitigation will require integrated, multisectoral strategies that combine improved sanitation and hygiene, nutrient-dense diets, and interventions to reduce zoonotic and environmental pathogen exposure through improved household and livestock management practices.

## Maternal influence

Maternal health and nutrition play a vital role in shaping the infant metabolome and can influence up to 30.3% of the risk for childhood stunting (158). Factors such as short pregnancy intervals, inadequate maternal weight gain, and infections during pregnancy significantly affect fetal metabolic development (159). Adequate maternal intake of amino acids, fatty acids, vitamins, and minerals is essential for proper fetal growth and metabolic programming, while deficiencies can lead to stunted fetal development and long-term health issues. Specifically, insufficient amino acids disrupt fetal metabolism (61), and a lack of omega-3 and omega-6 fatty acids can impair brain development and growth (160). Additionally, low maternal levels of trace elements like manganese, iron, zinc, iodine, and selenium are associated with higher risks of low birth weight and small-for-gestational-age infants (161).

Zinc and vitamin B12 deficiencies during pregnancy further exacerbate the risk of childhood stunting, as both nutrients are vital for fetal development. Zinc deficiency, which can arise from poor maternal diet or genetic factors such as ZIP4 mutations, impairs enzyme activity and immune function, limiting zinc availability in breast milk (162). Vitamin B12 deficiency disrupts key metabolic pathways, including taurine and hypotaurine metabolism, with taurine identified as a potential biomarker for B12 insufficiency (163). Evidence suggests that higher folate consumption may help mitigate stunting risks in children with B12 deficiency (164), and a study in Nepal showed that better maternal B12 status during pregnancy correlated with improved child height at 5 years old (165). Additionally, adequate maternal intake of one-carbon nutrients has been associated with enhanced cognitive development in offspring (166).

The “thrifty phenotype theory” (167) suggests that malnutrition around the time of conception induces fetal adaptations that, while aimed at survival, may predispose individuals to poor health and lower socioeconomic outcomes later in life. These maladaptive responses triggered by maternal, fetal, or placental stressors can impair fetal development and increase the risk of long-term metabolic disorders (168). Folate, essential for DNA synthesis and methylation, is particularly crucial during embryonic development. However, genetic variants like the 677CT polymorphism in the MTHFR gene can reduce folate bioavailability and raise homocysteine levels which can increase the risk of adverse fetal outcomes (169, 170). Additionally, maternal metabolic health such as insulin resistance linked to poor diet or obesity, can disrupt fetal glucose and lipid metabolism, contributing to fetal growth restriction and increased risk of stunting in early life (171). Chronic maternal inflammation or enteropathy may further compromise fetal development, as shown by elevated maternal sCD14 levels correlating with pro-inflammatory immune responses in stunted children (172).

Environmental factors further exacerbate metabolic disruptions in the infant. Exposure to environmental toxins, such as heavy metals and persistent organic pollutants, can impair nutrient transfer from mother to fetus, leading to metabolic imbalances that increase the risk of stunting (173). Additionally, maternal infections during pregnancy can trigger inflammatory responses that disrupt placental function and nutrient delivery, negatively impacting fetal metabolome development and increasing the likelihood of childhood stunting (174).

Studies highlight the strong connection between maternal and child undernutrition. Maternal metabolites can influence newborn size independently of maternal BMI and glycemia, emphasizing the critical role of maternal metabolic status (175). Additionally, approximately 50% of childhood stunting occurs in utero, with stunted mothers more likely to have smaller babies than their non-stunted counterparts (107, 176). Adequate maternal nutrition is essential to support infant growth and development (177). In low and middle-income countries, animal-source foods rich in essential amino acids are particularly crucial for linear growth and development, emphasizing the importance of sufficient maternal nutrition during pregnancy (178). Thus, ensuring optimal maternal nutrient intake, reducing metabolic stressors, and minimizing environmental risks can significantly improve infant health outcomes and help reduce the prevalence of childhood stunting.

**Figure 4:**
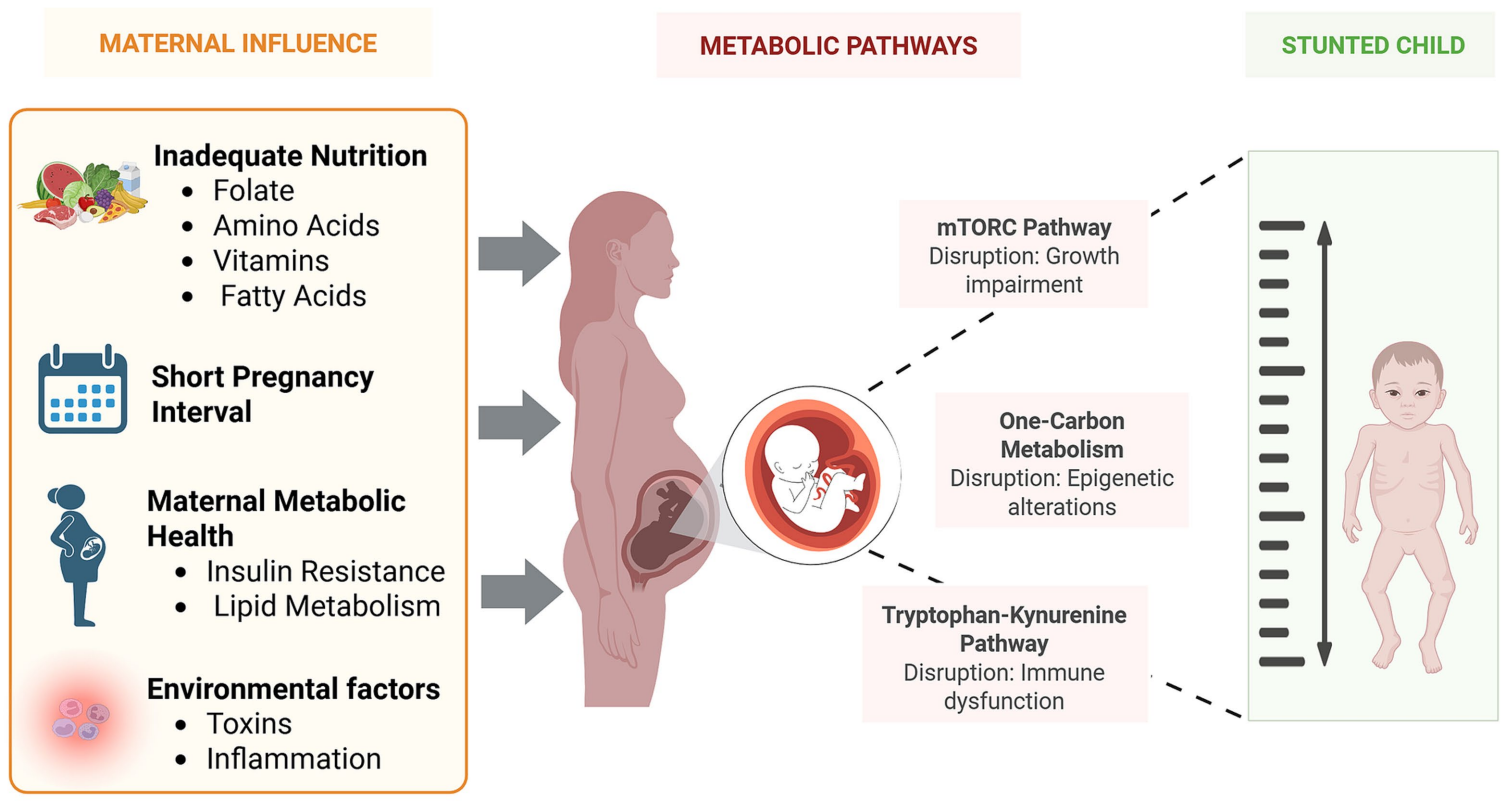
Integration of maternal factors and disrupted metabolic pathways contributing to growth faltering. Maternal factors such as inadequate nutrition, compromised metabolic health, and overall poor well-being can disrupt both maternal and fetal metabolic pathways, increasing the risk of childhood stunting. Created in BioRender. Crd, D. (2026), https://BioRender.com/hskmumh.

The interconnection of the several pathways previously discussed is summarized in Figure 4.

## Implications of metabolic pathways and biomarkers in the management of childhood stunting

The interplay between disrupted metabolic processes and nutritional deficiencies underscores the multifactorial nature of stunting. Identification of specific metabolites, such as those linked to amino acid metabolism, energy production, systemic inflammation and gut microbial activity, may provide deeper insights into the biological mechanisms contributing to growth impairment.

Based on the above discussions, the following strategies for alleviating childhood stunting may require further research:

1. Nutritional Interventions: Providing diets supplemented with important macronutrients and micronutrients, including essential and non-essential amino acids, vitamins (vitamin A, folate, vitamins B6 and B12) and minerals (zinc, iron, calcium, iodine and selenium) are critical to addressing deficiencies that impair growth and metabolism (179). These interventions may help address disruptions of biochemical pathways involving mTOR, Tryptophan-Kynurenine and OCM dysregulations.
2. Gut Health and Microbiota Restoration: Alterations in gut microbiota composition are strongly associated with stunting, making microbiota-targeted therapies an essential component of management (48, 132). Strategies to restore the gut and microbiome health merits more investigation.
3. Inflammation Reduction: Chronic systemic inflammation, often resulting from recurrent infections and poor sanitation, significantly contributes to stunting. Strategies such as improving WASH and reducing exposure to infectious agents are essential in managing inflammation (146). This intervention along with nutrient supplementation and microbiota restoration may address chronic inflammation, EED and microbiome disruption.
4. Maternal Centered Interventions: These are interventions that focus on improving maternal nutrition, metabolic health, and overall well-being to reduce the risk of childhood stunting. Ensuring that pregnant women receive a balanced diet rich in essential nutrients, including iron, folate, zinc, vitamin B12, and omega-3 fatty acids, is crucial for supporting fetal development. Micronutrient supplementation should be prioritized to prevent deficiencies that can impair growth (180, 181). Additionally, reduction of maternal inflammation through anti-inflammatory diets and gut health optimization can promote better fetal development and long-term health outcomes (182, 183). Proper pregnancy spacing allows maternal nutrient stores to replenish, leading to improved pregnancy outcomes (184), while infection control measures help prevent complications that contribute to stunting (185).
5. Integrated Public Health Approaches: It is important to address socioeconomic determinants of stunting through public health programs focused on maternal nutrition, antenatal care, and access to healthcare. These programs address the root causes of stunting while supporting child growth and development (186).

A holistic approach combining multiomics-derived nutritional and microbiota interventions, control of infection, reduction of inflammation as well as systemic public health efforts may offer an effective strategy for better management of childhood stunting.

## Conclusion

Childhood stunting results from a complex interaction of malnutrition, infections, maternal and socio-environmental factors, with interventions often yielding mixed results. The persistence of stunting despite these efforts highlights the need for a deeper understanding of its underlying mechanisms. Advanced techniques in systems biology like multiomics approaches offer insights into molecular disruptions such as mTOR inactivation, tryptophan-kynurenine pathway dysregulation, methylation dysfunction and microbiome disturbance among others are linked to malnutrition and inflammation. However, much of the current evidence is associative rather than causal, underscoring the need for longitudinal and mechanistic studies to validate biomarkers and therapeutic targets. Future research integrating multi-omics approaches with clinical and public health strategies may enable precision nutrition and targeted interventions capable of sustainably improving child growth outcomes.
